# Soluble Forms of Intercellular and Vascular Cell Adhesion Molecules Independently Predict Progression to Type 2 Diabetes in Mexican American Families

**DOI:** 10.1371/journal.pone.0151177

**Published:** 2016-03-23

**Authors:** Hemant Kulkarni, Manju Mamtani, Juan Peralta, Marcio Almeida, Thomas D. Dyer, Harald H. Goring, Matthew P. Johnson, Ravindranath Duggirala, Michael C. Mahaney, Rene L. Olvera, Laura Almasy, David C. Glahn, Sarah Williams-Blangero, Joanne E. Curran, John Blangero

**Affiliations:** 1 South Texas Diabetes and Obesity Institute, University of Texas Rio Grande Valley School of Medicine, Brownsville, TX, United States of America; 2 Department of Psychiatry, University of Texas Health Science Center at San Antonio, San Antonio, TX, United States of America; 3 Department of Psychiatry, Yale University School of Medicine, New Haven, CT, United States of America; 4 Olin Neuropsychiatric Research Center, Institute of Living, Hartford Hospital, Hartford, CT, United States of America; University of Catanzaro Magna Graecia, ITALY

## Abstract

**Objective:**

While the role of type 2 diabetes (T2D) in inducing endothelial dysfunction is fairly well-established the etiological role of endothelial dysfunction in the onset of T2D is still a matter of debate. In the light of conflicting evidence in this regard, we conducted a prospective study to determine the association of circulating levels of soluble intercellular adhesion molecule 1 (sICAM-1) and soluble vessel cell adhesion molecule 1 (sVCAM-1) with incident T2D.

**Methods:**

Data from this study came from 1,269 Mexican Americans of whom 821 initially T2D-free individuals were longitudinally followed up in the San Antonio Family Heart Study. These individuals were followed for 9752.95 person-years for development of T2D. Prospective association of sICAM-1 and sVCAM-1 with incident T2D was studied using Kaplan-Meier survival plots and mixed effects Cox proportional hazards modeling to account for relatedness among study participants. Incremental value of adhesion molecule biomarkers was studied using integrated discrimination improvement (IDI) and net reclassification improvement (NRI) indexes.

**Results:**

Decreasing median values for serum concentrations of sICAM-1 and sVCAM-1 were observed in the following groups in this order: individuals with T2D at baseline, individuals who developed T2D during follow-up, individuals with prediabetes at baseline and normal glucose tolerant (NGT) individuals who remained T2D-free during follow-up. Top quartiles for sICAM-1 and sVCAM-1 were strongly and significantly associated with homeostatic model of assessment—insulin resistance (HOMA-IR). Mixed effects Cox proportional hazards modeling revealed that after correcting for important clinical confounders, high sICAM-1 and sVCAM-1 concentrations were associated with 2.52 and 1.99 times faster progression to T2D as compared to low concentrations, respectively. Individuals with high concentrations for both sICAM-1 and sVCAM-1 progressed to T2D 3.42 times faster than those with low values for both sICAM-1 and sVCAM-1. The results were similar in women in reproductive age group and the remainder of the cohort. Inclusion of sICAM-1 and sVCAM-1 in predictive models significantly improved reclassification and discrimination. The majority of these results were seen even when the analyses were restricted to NGT individuals.

**Conclusion:**

Serum concentrations of sICAM-1 and sVCAM-1 independently and additively predict future T2D and represent important candidate biomarkers of T2D.

## Introduction

Vascular endothelial dysfunction is a characteristic feature of type 2 diabetes. An increasing number of studies have shown that the serum concentrations of the adhesion molecules involved in leukocyte adhesion to the endothelial surface are elevated in individuals with T2D as compared to healthy counterparts.[1–8] It appears that the relationship between endothelial dysfunction and T2D may be bidirectional, such that T2D can lead endothelial dysfunction [9, 10] and that endothelial dysfunction can lead to T2D.[11, 12] However, evidence for endothelial dysfunction preceding or correlating with the risk of later T2D is inconclusive.

Meigs and coworkers showed in two large epidemiological studies (using data from the Nurses’ Health Study (737 incident cases of type 2 diabetes and 785 controls) [13] and from Framingham Offspring Study (2,011 diabetes-free individuals) [14]) that adhesion molecules that signify endothelial dysfunction independently predict the risk of future T2D. Similarly, based on data from the Women’s Health Initiative Observational Study (WHIOS; 1,584 incident diabetes cases matched with 2,198 controls), Song et al [15] independently replicated the association of circulating adhesion molecules with risk of incident T2D. Finding from Thorand et al [16], Stranges et al [17] and Rossi et al (using flow mediated dilation to measure endothelial dysfunction) [18] also support a possible etiological role of endothelial dysfunction in T2D. While the molecular mechanisms for this that directly link endothelial dysfunction and T2D are unclear, it has been posited [19–22] that endothelial dysfunction may induce insulin resistance and thereby facilitate progression to T2D. This is partly attributable to the endocrine role of the adipose tissue since reversion of the adiponectin:leptin ratio correlates with both insulin resistance and endothelial dysfunction.[23, 24] It has also been argued that decreased nitric oxide availability observed in a state of insulin resistance has been correlated with vascular cellular adhesion molecule-1 (VCAM-1) levels.[25]

Yet, the relationship between endothelial dysfunction and subsequent T2D has not been universally observed. Using data from the Framingham Heart Study, Dallmeier and others [26] recently showed that while intercellular adhesion molecule 1 (ICAM-1) concentration in serum is increased in individuals who eventually develop T2D, this association does not hold after accounting for other clinical covariates commonly used to predict T2D (e.g. age, sex, cohort, body mass index, fasting glucose, systolic blood pressure, high-density lipoprotein cholesterol, triglycerides, and smoking). Similarly, using a subset of data from the WHIOS, Chao et al [27] found that addition of ICAM-1 to traditional risk factors of T2D does not improve prediction. Thus, while there is evidence for an etiological role of endothelial dysfunction in T2D, it is unclear if this relationship is primary, is related to the exact adhesion molecule measures or is secondary to clinical comorbidities.

It is possible that ethnic heterogeneity could explain the variability of results and conclusion across epidemiological studies. Currently, there is increasing evidence to demonstrate that endothelial oxidative stress, inflammatory biomarkers and response to L-arginine supplementation can all differ by race.[28–31] We conducted a prospective study of progression to T2D in initially T2D-free individuals of Mexican American origin in San Antonio. Mexican Americans represent a minority ethnic group in the United States that is at a high risk of type 2 diabetes (reference). Data for this study come from the San Antonio Family Heart Study (SAFHS) which enrolled participants from large and extended Mexican American families.[32–34] Using these data, we tested the hypothesis that circulating levels of soluble intercellular adhesion molecule 1 (sICAM-1) and soluble vascular cell adhesion molecule 1 (sVCAM-1) are independently and additively predictive of future T2D.

## Materials and Methods

### Study Participants

**Fig 1:**
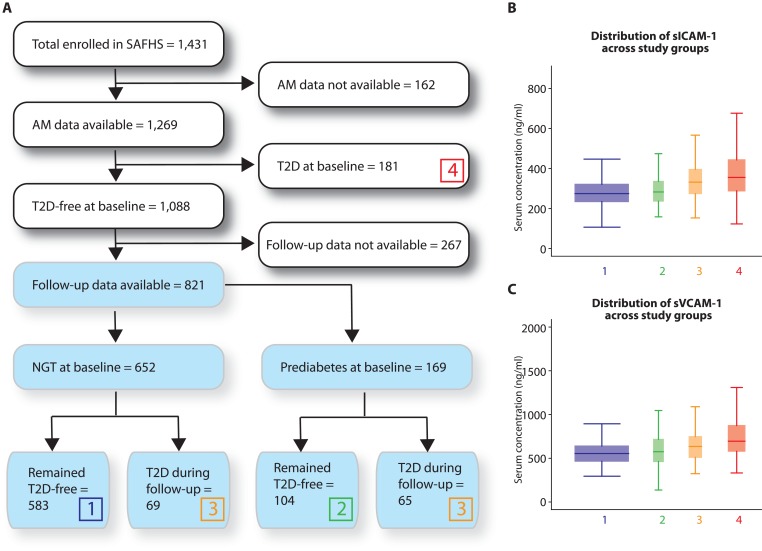
Description of the study cohort and distribution of the serum concentration of adhesion molecules in study participants. **(A)** Selection criteria. White colored boxes indicate the cross-sectional component of the study while the blue colored boxes indicate the prospective component of the study. Color-coded numbered boxes represent the four study groups for which distribution of adhesion molecules is given in panels B and C. AM, adhesion molecules **(B and C)** Box and whisker plots showing the distribution of sICAM-1 (B) and sVCAM-1 (C) concentration in serum. The width of the boxes is proportional to the number of individuals within the group. The boxes are color-coded to match the numbered boxes in panel A.

We used data from the Mexican American families recruited in the SAFHS.[32–34] The Institutional Review Board of the University of Texas Health Science Center at San Antonio approved the study and a written informed consent was obtained from all the study participants. At baseline, this cohort consisted of 1,431 individuals from 42 large and extended pedigrees. We conducted the analyses using the cross-sectional arm of the study (white boxes in Fig 1A) as well as the prospective arm of the study (blue boxes in Fig 1A). The cross-sectional arm of the present study included a total of 1,269 individuals on whom sICAM-1 and sVCAM-1 data was available. We found that 267 (24.5%) of the initially T2D-free individuals did not appear fir any follow-up visit later and had to be excluded from longitudinal analyses. The clinical characteristics of those who were lost to follow-up were not statistically significantly different from those who were included (data not shown). For the longitudinal arm of the study, we included a total of 821 individuals without diabetes at baseline who were followed for up to three additional visits spaced approximately 5 years apart (9752.95 person-years of follow-up with a maximum follow-up of 23.53 years). The protocol for selection of the study participants is shown in Fig 1A.

### Phenotypic Data

We included phenotypic data on the following important predictors of type 2 diabetes: age, sex, waist circumference, body mass index, systolic and diastolic blood pressure, fasting and 2-hour plasma glucose, fasting insulin, total serum cholesterol, serum triglycerides, high-density lipoprotein (HDL) cholesterol and use of lipid-lowering and anti-hypertensive drugs. Methods used to measure these variables have been described in details previously. [32–34] Briefly, plasma glucose and insulin levels were measured at fasting and 2 h after administration of 75 g oral glucose, as detailed in [34]. Type 2 diabetes was diagnosed using by the diagnostic criteria of the American Diabetes Association Clinical Practice Recommendations 2004 (fasting plasma glucose level ≥126 mg/dl [7.0 mmol/l], plasma glucose ≥200 mg/dl [11.1 mmol/l] at 2 h after oral glucose challenge, or both).[35] Also, individuals were considered to have diabetes if they reported use of antidiabetic medication.[36] For all the participants who reported T2D during follow-up we had information on the date of T2D diagnosis which was used for censoring. Homeostasis model assessment of insulin resistance (HOMA-IR) values were calculated from fasting glucose and insulin measures according to the formula (fasting glucose [mmol/l] × fasting insulin [μU/ml]/22.5).[37] Prediabetes was defined as presence of either impaired fasting glucose (fasting plasma glucose 100–125 mg/dl [4.56–7 mmol/l]) or impaired glucose tolerance (2 hour post-prandial plasma glucose 140–199 mg/dl [7.78–11.0 mmol/l]) or both. sICAM-1 and sVCAM-1 concentrations were measured in serum samples (diluted 1:20) using an enzyme-linked immunosorbent assay (R&D Systems, Minneapolis, MN). Measurement of adhesion molecules was based on the same fasting samples used for measurement of glucose, insulin and other biochemical markers.

### Statistical Analysis

#### Survival analyses

Our primary outcome of the study was development of incident T2D. We conducted survival analyses graphically using Kaplan-Meier survival plots. We then estimated mixed effects Cox proportional hazards (PH) which differs from the classical Cox proportional hazards modeling as mixed effects hazards account for relatedness in family studies. Explicitly, a mixed effects Cox PH model is of the form: [38, 39]

$$\lambda (t)=\lambda_{0}(t)e^{X\beta +Zb},$$

where λ is the hazard function; λ_0_ the unspecified baseline hazard; X and Z are covariate matrices for fixed and random effects, respectively; and β and b represent the regression coefficient vectors associated with fixed and random effects, respectively. In this equation the random effects are assumed to be normally distributed as N(0,Σ(θ)) where Σ represents a variance matrix of parameters θ. In the case of family studies, the random effects are approximated by the kinship among pairs of individuals. We used this approach to conduct all the multivariable regression modeling in this study. All mixed effects Cox PH models included the following covariates as fixed effects: age, sex, age*sex interaction, waist circumference, body mass index, systolic and diastolic blood pressures, total serum cholesterol, serum triglycerides, serum HDL-cholesterol, use of anti-lipid medications and use of antihypertensive medication.

#### Distributional descriptions

We used box and whisker plots to describe the distributions of study variables. Continuous variables were also described using the mean and its standard error. To compare median values of a continuous variable across categories of a categorical variable we used Mann-Whitney U (2 categories) or Kruskal-Wallis (3 categories) tests.

#### Improvement in predictive performance

We used the integrated discrimination improvement (IDI) and net reclassification improvement (NRI) indexes to quantify the contribution of adhesion molecules at baseline to prediction of future T2D. [40] These indexes quantify the ability of a marker to better discriminate (as compared to a base model) between cases and non-cases and to better reclassify the cases and non-cases to appropriate groups, respectively.

## Results

### Characteristics of Study Participants

For the longitudinal arm of the study we included a total of 821 initially T2D-free individuals on whom data for sICAM-1, sVCAM-1, follow-up and all phenotypic traits was available. The clinical characteristics of these individuals at baseline are described in Table 1. The participants were young with a majority of females. Both central and general obesity was common (~42% and 35%, respectively) but the prevalence of hypertension was low (9%). Over 20% individuals had prediabetes at baseline. Lipid profile of the study participants did not reveal any major abnormalities. Very few participants were receiving lipid-lowering and antihypertensive drugs (<2% and ~5%, respectively). A total of 134 individuals (16.32%) developed T2D during follow-up.

**Table 1:** Clinical characteristics of the SAFHS included in the longitudinal arm of the study.

Characteristic	SAFHS cohort(n = 821)*
Age at enrolment (y)	35.39 (0.51)
Average length of follow-up (y)	11.88 (0.13)
Females	511 (62.24)
Waist (cm)	92.53 (0.61)
Central obesity (Waist circumference ≥102 cm for males and ≥88 cm for females)	343 (41.78)
Body Mass Index (BMI, Kg/m^2^)	28.86 (0.23)
Obesity (BMI ≥30 Kg/m^2^)	288 (35.08)
Systolic blood pressure (SBP, mmHg)	117.0 (0.58)
Diastolic blood pressure (DBP, mmHg)	70.2 (0.34)
Hypertension (SBP≥140 mmHg and/or DBP≥90mmHg)	75 (9.14)
Fasting glucose (mmol/l)	4.86 (0.02)
2-hour post challenge glucose (mmol/l)	5.67 (0.06)
Prediabetes at baseline	169 (20.58)
Impaired fasting glucose only	51 (6.21)
Impaired glucose tolerance only	81 (9.87)
Impaired fasting glucose and impaired glucose tolerance	37 (4.50)
Total serum cholesterol (mg/dl)	187.32 (1.34)
Serum triglycerides (mg/dl)	138.69 (4.57)
HDL cholesterol (mg/dl)	51.12 (0.45)
Participants taking lipid lowering medication	13 (1.58)
Participants taking anti-hypertensive medications	44 (5.36)

*, numbers indicate mean(SE) for continuous variables and n (%) for categorical variables

### Distribution of sICAM-1 and sVCAM-1

As can be seen from Fig 1B and 1C, the distribution of sICAM-1 and sVCAM-1 displayed interesting patterns. For example, highest median values for these adhesion molecules (356.01 ng/ml and 695.26 ng/ml, respectively) were observed for individuals who had T2D at baseline (group 4 in Fig 1B and 1C) followed by individuals who developed T2D during follow-up (group 3; 332.16 ng/ml and 635.86 ng/ml, respectively). Next, those with prediabetes at baseline but who did not develop T2D during follow-up (group 2) had even lower concentrations of the adhesion molecules (283.19 ng/ml and 575.94 ng/ml, respectively) but the lowest median concentrations were observed for those individuals who had normal glucose tolerance (NGT, group 1; 274.74 ng/ml and 553.64 ng/ml, respectively). Compared to group 1 the sICAM concentrations in groups 3 and 4 were highly significantly different (Mann-Whitney test p = 7.13x10^-11^ and <1x10^-22^, respectively) but those in group 2 did not differ significantly from group 1 (p = 0.1466). Similarly, the Mann-Whitney test significance values for difference in median concentration of sVCAM in groups 2, 3 and 4 compared to group 1 were 0.2690, 1.46x10^-7^ and <1x10^-22^,respectively.

### Association of sICAM-1 and sVCAM-1 with Insulin Resistance

We investigated if the HOMA-IR values differed significantly based on categories of serum concentration of adhesion molecules. To simplify the use of sICAM-1 and sVCAM-1 concentrations for practical use, we dichotomized the study participants into high and low values based on sICAM-1 and sVCAM-1 concentrations. The high value group represented the top quartile of the distribution while the low value group was a conglomeration of the lower three quartiles. The cutoff points used for high values of sICAM-1 and sVCAM-1 were >336.21 ng/ml and >670.42 ng/ml, respectively. To examine if these two adhesion molecules exhibit a potentially independent and additive influence on the risk of future T2D, we created a three-category variable. The categories were: low values for both sICAM-1 and sVCAM-1, high value for either sICAM-1 or sVCAM-1 and high values for both aICAM-1 and sVCAM-1.

The results are shown in Table 2. We observed that with the exception of sVCAM-1 categories in the NGT individuals, the categories of sICAM-1 and sVCAM-1 concentrations were consistently and significantly associated with insulin resistance as measured by HOMA-IR. High values of sICAM-1 and sVCAM-1 concentrations were associated with high values of HOMA-IR and the pattern was even more clearly seen for the variable that combined sICAM-1 and sVCAM-1 concentrations—median HOMA-IR dropped from 3.28 for the High/High group to 1.99 for the Low/Low group for the entire cohort and from 2.35 to 1.87 for NGT individuals.

**Table 2:** Median HOMA-IR values based on serum concentrations of sICAM-1 and sVCAM-1.

Biomarker and categories	Median HOMA-IRAll individuals	Median HOMA-IRNGT individuals
sICAM-1		
High	2.90	2.35
Low	2.04	1.90
Mann-Whitney p	4.08x10^-9^	0.0012
sVCAM-1		
High	2.67	2.15
Low	2.08	1.94
Mann-Whitney p	3.20x10^-5^	0.1121
Combined groups (sICAM-1/sVCAM-1)		
High/High	3.28	2.35
High/Low or Low/High	2.53	2.26
Low/Low	1.99	1.87
Kruskal-Wallis p	3.04x10^-9^	0.0096

### Prediction of T2D Based on sICAM-1 and sVCAM-1

**Fig 2:**
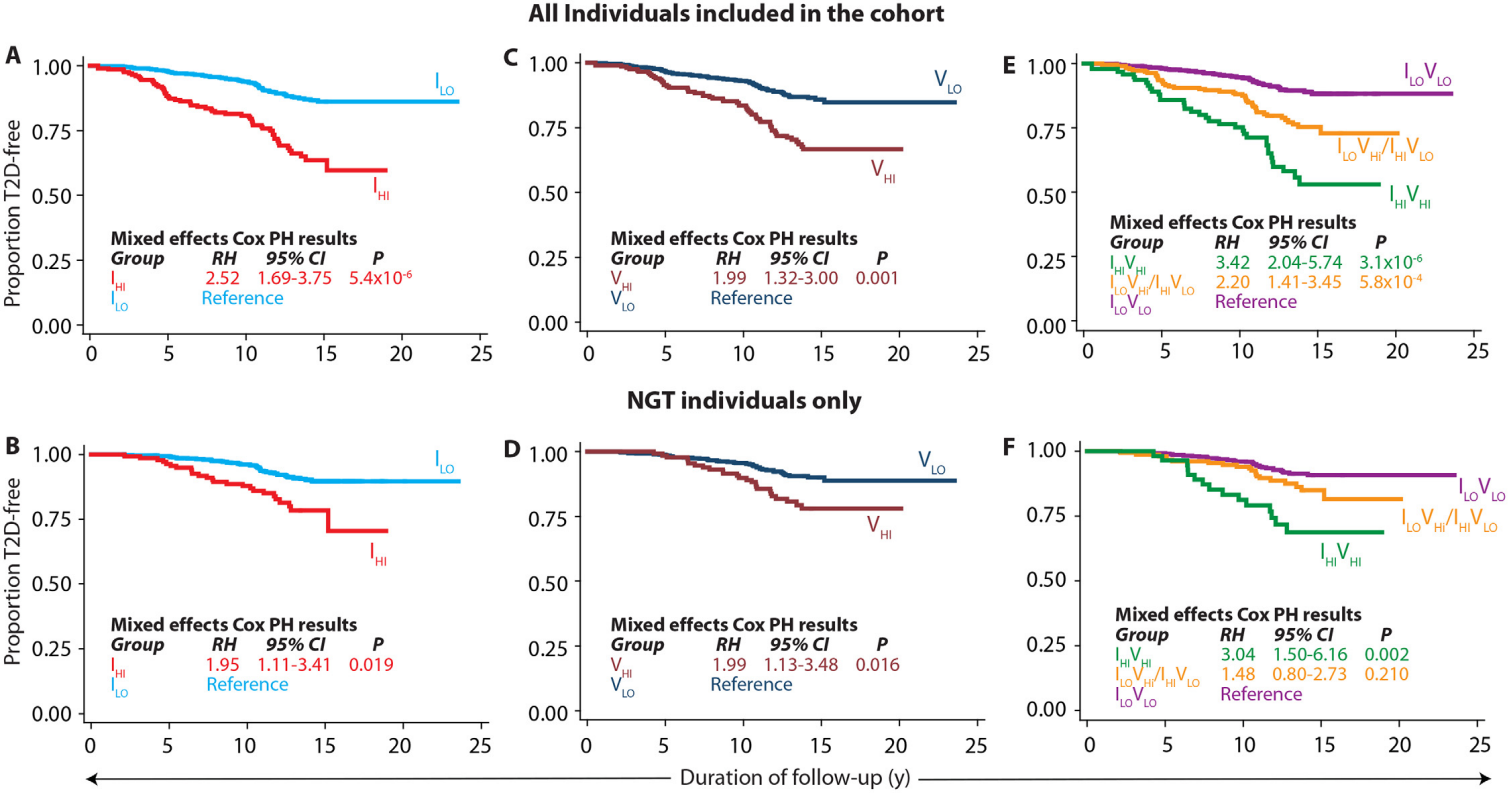
Survival analyses for time to onset of type 2 diabetes from the day of enrolment into the study based on the serum concentrations of adhesion molecules. **(A and B)** Association of sICAM-1 concentration with incident type 2 diabetes, **(C and D)** association of sVCAM-1 concentration with incident type 2 diabetes, and **(E and F)** association of a combination of the serum concentrations of sICAM-1 and sVCAM-1 with incident type 2 diabetes. The group identifiers are as follows: I_LO_, sICAM-1 ≤ 336.21 ng/ml; I_HI_, sICAM-1 > 336.21 ng/ml; V_LO_, sVCAM-1 ≤ 670.42 ng/ml; V_HI_, sVCAM-1 > 670.42 ng/ml. Panels E and F use a combination of these groups as indicated in the Fig. All panels show Kaplan-Meier survival plots for the indicated groups. Inset in each panel are results from mixed effects Cox proportional hazards regression. All regression models used following variables as fixed effects covariates in addition to kinship as random effects: age, sex, age*sex interaction, waist circumference, body mass index, systolic and diastolic blood pressures, total serum cholesterol, serum triglycerides, serum HDL-cholesterol, use of anti-lipid medications and use of antihypertensive medication. PH, proportional hazards; RH, relative hazards; CI, confidence interval; NGT, normal glucose tolerance.

We then conducted survival analyses to examine the predictive role of the circulating adhesion molecules in incident T2D. Kaplan-Meier plots for progression to T2D based on independent and combined groups of sICAM-1 and sVCAM-1 concentrations are shown in Fig 2. These results clearly showed that individuals in the top quartile group for both sICAM-1 (Fig 2A) and sVCAM-1 (Fig 2C) progressed to T2D more rapidly than the respective reference groups. Moreover, the influence of these two adhesion molecules was additive as depicted by an even faster progression to T2D when an individual had high values for both sICAM-1 and sVCAM-1 (Fig 2E). These results were seen even in the subset of individuals who had NGT at baseline (Fig 2B, 2D and 2F). The results of multivariable mixed effects Cox PH models for the corresponding analyses demonstrated that indeed, independent of the common clinical predictors of future T2D, high values of sICAM-1 and sVCAM-1 were significantly predictive of incident T2D –singly or in combination. For example, while the individuals with high sICAM-1 and high sVCAM-1 progressed 2.52 and 1.99 times faster than the corresponding reference groups, those who had high values for both sICAM-1 and sVCAM-1 progressed 3.42 times faster to T2D as compared to those with low values for both sICAM-1 and sVCAM-1. With the exception of the group representing a high value for either sICAM-1 or sVCAM-1 concentration, all the results observed the entire cohort were also observed in the NGT subset of the cohort.

### Do Menstruation-Related Variations in Circulating Adhesion Molecules Influence Prediction of T2D?

We investigated the possibility that our results may have been influenced by variations in circulating adhesion molecule concentrations across phases of menstrual cycles. We did not have data on the exact date of last menstrual period. Therefore, we conducted the statistical analyses on two subsets of the original cohort—women in reproductive age group and the remainder of the cohort (which included women ≥45 years of age and all males). Notably, only 12.69% of the women in reproductive age group progressed to T2D while 19.54% of the remainder of the cohort progressed to T2D during follow-up. To study the association of circulating adhesion molecules with T2D stratified by the reproductive status of women, we conducted mixed effects Cox regression. Table 3 shows the results of these analyses. We found that in both the abovementioned subsets of the cohort the association categorized on the basis of sICAM-1 and sVCAM-1 concentrations (as defined in Fig 2E) was statistically significant compared to the reference group. This association could be seen before as well as after adjustment for several other clinically relevant confounders listed in Table 3. Further, the test for heterogeneity of the relative hazards across these two subsets was not significant indicating that women in reproductive age group did not account for the observed associations. Rather, the observations were consistent irrespective of gender and age. These results indirectly affirm the hypothesis that menstruation-related variations in adhesion molecule concentrations were an unlikely driver of the results we observed.

**Table 3:** Mixed effects Cox regression for prediction of incident type 2 diabetes in women in reproductive age group (WRAG) and the remainder of the cohort.

Group	Women in reproductive age group (n = 386)		Remainder of the cohort (n = 435)		P_het_**
	UnadjustedRH (95% CI), p	Adjusted*RH (95% CI), P	UnadjustedRH (95% CI), P	Adjusted*RH (95% CI), p	
I_LO_V_LO_	Ref	Ref	Ref	Ref	--
I_LO_V_HI_/I_HI_V_LO_	3.30 (1.62–6.34), 0.0009	2.46 (1.20–5.00), 0.0130	2.16 (1.26–3.71), 0.0050	1.99 (1.11–3.56), 0.0210	0.944
I_HI_V_HI_	8.61 (3.91–19.0),9.2x10^-8^	3.51 (1.59–7.77), 0.0019	3.93 (2.14–7.18), 8.8x10^-6^	3.39 (1.77–6.52), 0.0003	0.653

*, All models used following variables as fixed effects covariates in addition to kinship as random effects: age, sex, age*sex interaction, waist circumference, body mass index, systolic and diastolic blood pressures, total serum cholesterol, serum triglycerides, serum HDL-cholesterol, use of anti-lipid medications and use of antihypertensive medication.

**, test of heterogeneity of the adjusted model results across the two subsets.

### Improvement in Clinical Prediction of T2D

Considering the independent and additive influence of sICAM-1 and sVCAM-1 concentrations on progression to T2D, we next determined whether addition of these biomarkers to routinely used clinical predictors of T2D leads to a significant improvement in clinical prediction. For this, we estimated the IDI and NRI indexes. Table 4 shows the results of these analyses. Our results indicate that singly or together these two biomarkers significantly improve the net reclassification for the entire cohort as well as for the NGT individuals (p values ranging from 0.0072–2.03x10^-10^). Discrimination between progressors and non-progressors to T2D was statistically significant when examined for the entire cohort but not when restricted to NGT individuals. In the entire cohort, the combined use of sICAM-1 and sVCAM-1 improved discrimination by ~3.5% (p = 0.0002) but in the NGT individuals this improvement was ~1.5% which was marginally non-significant (p = 0.0729).

**Table 4:** Improved clinical prediction of incident type 2 diabetes based on sICAM-1 and sVCAM-1 as independent biomarkers*.

Biomarker**	IDI (95% CI), p	NRI (95% CI), p
All individuals		
sICAM-1	0.0245 (0.0098–0.0392), 0.0012	0.5482 (0.3595–0.7369), 1.27x10^-8^
sVCAM-1	0.0195 (0.0062–0.0328), 0.0043	0.4680 (0.2793–0.6567), 1.19x10^-6^
Combination	0.0346 (0.0166–0.0526), 0.0002	0.6127 (0.4240–0.8014), 2.03x10^-10^
NGT individuals		
sICAM-1	0.0049 (-0.0049–0.0147), 0.3291	0.3493 (0.0947–0.6039), 0.0072
sVCAM-1	0.0131 (0.0002–0.0260), 0.0485	0.3796 (0.1250–0.6342), 0.0035
Combination	0.0146 (-0.0013–0.0305), 0.0729	0.4005 (0.1459–0.6551), 0.0021

*, all the results are based on comparison of a model that included the indicated biomarker in addition to the covariates included in the base model. The base model included following covariates: age, sex, age*sex interaction, waist circumference, body mass index, systolic and diastolic blood pressures, total serum cholesterol, serum triglycerides, serum HDL-cholesterol, fasting glucose, use of anti-lipid medications and use of antihypertensive medication

**, biomarker coding: sICAM-1 –serum sICAM-1 concentration >336.21 ng/ml is coded as 1 else 0; sVCAM-1—serum svCAM-1 concentration >670.42 ng/ml is coded as 1 else 0; Combination—serum sICAM-1 concentration >336.21 ng/ml and serum svCAM-1 concentration >670.42 ng/ml coded as 2, either serum sICAM-1 concentration >336.21 ng/ml or serum svCAM-1 concentration >670.42 ng/ml coded as 1, and serum sICAM-1 concentration ≤336.21 ng/ml and serum svCAM-1 concentration ≤670.42 ng/ml coded as 0

IDI, integrated discrimination improvement; NRI, continuous version of the net reclassification improvement; CI, confidence interval; p, significance value for the null hypothesis of no improvement; NGT, normal glucose tolerance

## Discussion

Accumulation of soluble adhesion molecules in the serum is a result of proteolytic cleavage of the adhesion molecules bound to the endothelial cell membranes and their consequent release into circulation.[41–43] Therefore serum levels of adhesion molecules are considered to be an indirect measure of endothelial dysfunction. Our findings suggest that soluble versions of ICAM-1 and VCAM-1 are independent and additive biomarkers of incident T2D in Mexican Americans. We observed a clear association of the serum concentrations of these biomarkers with insulin resistance and future T2D. Further, inclusion of these biomarkers into predictive models improved reclassification and discrimination in most instances. The fact that the majority of the results were also observed in the NGT individuals points towards the possibility that endothelial dysfunction may exist years before clinically detectable dysglycemia occurs. Combined with the observation that the highest median values for sICAM-1 and sVCAM-1 were observed in individuals who had T2D at baseline, our study affords an indirect but significant support to the notion that the relationship between T2D and endothelial dysfunction is likely bidirectional. Our results thus agree with the body of literature that supports a contributory role of endothelial dysfunction in type 2 diabetes.

There are two interesting implications of our results in the light of our previous studies. First, we have shown that in healthy adults, smoking is strongly associated with serum concentration of adhesion molecules.[44] On the other hand, smoking—even passive smoking—is a known risk factor for type 2 diabetes.[45, 46] Our results thus provide a potential explanation for the etiological role of smoking in type 2 diabetes. It is conceivable that the smoking increases the risk of T2D by inducing insulin resistance through endothelial dysfunction. This pathway is also supported by the emerging association between smoking and growth differentiation factor-15 [47, 48]–another marker of endothelial dysfunction. Future studies need to test these hypotheses. Second, we have shown previously that there is a significant genetic correlation between sICAM-1 concentration and type 2 diabetes.[49] This finding assumes importance in the light of our observations that the adhesion molecule concentrations in the serum can predict future type 2 diabetes. These observations beckon an examination of the common genetic influences on endothelial dysfunction and type 2 diabetes. Genetic studies such as these can provide novel drug targets for prevention of T2D.

Some limitations of our study need to be considered before generalizing the results. First, our study was based on the high-risk Mexican American individuals and the results should not be readily applied to other populations. Second, even though our study found strong association of the adhesion molecules with insulin resistance, we cannot provide a direct experimental or mechanistic link between these clinical entities. Third, we did not have data on the exact date of last menstrual period which could have helped us directly estimate the putative influence of phases of menstrual cycle on the association of circulating adhesion molecules with incident T2D. This limitation is important in the light of the growing view that concentrations of circulating adhesion molecules vary by phase of menstrual cycle.[50–53] However, we believe that such an influence, even if operational, was unlikely to have confounded our results for the following two reasons: 1. Even after excluding the women in reproductive age groups from analyses (as shown in Table 3), the remainder of the cohort continued to show the associations with comparable strength and direction of associations. 2. Using the same data on adhesion molecules, we have previously demonstrated a strong genetic control over the circulating levels as well as their significant genetic correlation with insulin resistance.[44, 49, 54] It is conceivable that if the findings were to be confounded by phases of menstruation then the genetic signals observed previously would have obscured.

Notwithstanding these limitations, our study has the strengths of a prospective cohort, single ethnic background, family setting and long follow-up. In conclusion, we show that circulating levels of sICAM-1 and sVCAM-1 are independently and additively predictive of incident T2D. In the continued search for better and more informative biomarkers for T2D, sICAM-1 and sVCAM-1 represent important candidate biomarkers of T2D that need to be investigated in future studies.

## Supporting Information

S1 Data (XLSX)
